# Graph Structure-Based Simultaneous Localization and Mapping Using a Hybrid Method of 2D Laser Scan and Monocular Camera Image in Environments with Laser Scan Ambiguity

**DOI:** 10.3390/s150715830

**Published:** 2015-07-03

**Authors:** Taekjun Oh, Donghwa Lee, Hyungjin Kim, Hyun Myung

**Affiliations:** Urban Robotics Laboratory (URL), Korea Advanced Institute of Science and Technology (KAIST), 291 Daehak-ro (373-1 Guseong-dong), Yuseong-gu, Daejeon 305-701, Korea; E-Mails: buljaga@kaist.ac.kr (T.O.); leedonghwa@kaist.ac.kr (D.L.); hjkim86@kaist.ac.kr (H.K.)

**Keywords:** laser scan ambiguity, graph-based SLAM, intrinsic and extrinsic camera calibration, hybrid method

## Abstract

Localization is an essential issue for robot navigation, allowing the robot to perform tasks autonomously. However, in environments with laser scan ambiguity, such as long corridors, the conventional SLAM (simultaneous localization and mapping) algorithms exploiting a laser scanner may not estimate the robot pose robustly. To resolve this problem, we propose a novel localization approach based on a hybrid method incorporating a 2D laser scanner and a monocular camera in the framework of a graph structure-based SLAM. 3D coordinates of image feature points are acquired through the hybrid method, with the assumption that the wall is normal to the ground and vertically flat. However, this assumption can be relieved, because the subsequent feature matching process rejects the outliers on an inclined or non-flat wall. Through graph optimization with constraints generated by the hybrid method, the final robot pose is estimated. To verify the effectiveness of the proposed method, real experiments were conducted in an indoor environment with a long corridor. The experimental results were compared with those of the conventional GMappingapproach. The results demonstrate that it is possible to localize the robot in environments with laser scan ambiguity in real time, and the performance of the proposed method is superior to that of the conventional approach.

## 1. Introduction

For a robot to carry out tasks independently, it must know where it is located on the world model. This problem is referred to as SLAM (simultaneous localization and mapping) in the robotics society. With rising interest in the SLAM problem, a fundamental issue for exploration by robots in unknown environments, SLAM algorithms recently have been actively studied [1–22].

In efforts to solve the robot localization problem, a variety of sensors have been used, and SLAM algorithms using these sensors are employed. A representative approach is the use of a laser scanner [4–6]. In [4], a Rao–Blackwellized particle filter-based SLAM algorithm referred to as GMappingwas proposed, and the problem of particle depletion arising in particle filter-based SLAM was solved. Gouveia *et al.* proposed a multi-threaded GMapping technique for multi-processor computer architectures instead of a single thread [5]. This method exploits an increased number of particles for enhanced localization accuracy by performing the GMapping in parallel. In [6], a scan matching method and a multi-resolution likelihood map generation algorithm were used for UGV (unmanned ground vehicle) localization in large-scale indoor environments. This method reduces the searching scope in the process of scan matching. When the position of the robot is estimated by using a laser scanner, it is possible to precisely determine the position depending on the accuracy of the laser scanner. However, it is difficult to estimate the robot pose in environments such as a long corridor without any post or corner information, because the depth information obtained from the laser scanner does not change over time and is featureless.

Another method of estimating the pose of the robot involves the use of a camera [7,8]. Davison *et al.* proposed a method called MonoSLAM [7], which is performed using a single camera. MonoSLAM exploits a method called an active approach to carry out localization. This approach utilizes a general camera motion model designed under the assumption that the camera movement is smooth. However, because this approach uses only one camera, it is insufficient to solve the scale problem: the scale cannot be mathematically solved due to the lack of variables. Thus, in order to solve this problem practically, MonoSLAM should start with an object whose size is already known. In [8], a graph optimization technique and a monocular visual SLAM were exploited. A new loop closure method was proposed to practically resolve the scale drift resulting in a scale change caused by the scale problem. Still, there is an inherent problem in using the camera: the camera is sensitive to illumination changes, and errors may occur as a result in the robot localization.

As well as using a single sensor, localization has been performed by sensor fusion or 2D features in combination with depth data [9–12]. Ramos *et al.* proposed a conditional random field as a matching method where 2D laser scanner data are matched with feature data from a vision sensor [9]. However, it has been argued that it is ineffective for real-time operation, because of its computational burden.

May *et al.* proposed a 3D mapping algorithm for a TOF (time-of-flight) camera, by employing SIFT (scale-invariant feature transform) matching [23] from the TOF camera's image data and ICP (iterative closest point) from the TOF camera's depth data [10]. In order to increase the robustness, filtering and a calibration method were proposed. However, the TOF camera inherently has the disadvantage of sparsity of depth data.

Zhang *et al.* proposed a method called line-based EKF (extended Kalman filter) SLAM using a camera and a 2D laser scanner [11]. This method uses only a fixed ROI (region-of-interest) of the image obtained from the camera. Because the ROI is not changed, however, this method cannot cope with various circumstances. In this method, line segments in the image are extracted using the Sobel filter and morphological image processing technique. The line segments from the camera image and laser scanner depth data are fused in accordance with the Bayesian decision to reject outlier line segments. EKF-based SLAM performed via this method uses a total of three EKFs. The first filter estimates the robot pose from camera data. The second estimates the pose from laser range data. The last is for updating the data obtained from each EKF. However, the time to process a frame is two seconds, because of the massive calculations in the algorithm. This algorithm thus must be run offline, and real-time operation is difficult.

Henry *et al.* proposed a 3D mapping algorithm in indoor environments using a Kinect-style depth camera [12]. For 2D feature extraction, a combination of FAST feature detector [24] and a Calonder feature descriptor [25] is used to reduce the computation time. Furthermore, for frame-to-frame alignment and loop-closure detection, ICP and RANSAC (random sample consensus) [26] algorithms are used. However, the Kinect-style depth camera has a poor depth resolution and a smaller angular field of view than the laser scanner, which hinders the applicability to spaces with sensor data ambiguity.

This paper presents a graph structure-based SLAM using a 2D laser scanner and a monocular camera to solve the problems discussed above. The image feature data are acquired from a camera, and the depth information is acquired from a laser scanner. The shortcomings of each sensor are compensated by using a graph structure that fuses the obtained sensor data. This hybrid method integrating a laser scanner and a camera allows us to estimate the robot pose in environments with laser scan ambiguity where the pose estimation is difficult when solely using a laser scanner. Such environments are a long corridor or a significantly big space where the laser scanner can detect only one side of the surroundings because the range from the robot to the wall is larger than the maximum detectable range of the laser scanner. The hybrid method can overcome the disadvantages of each sensor. By using these methods, it is possible to accurately estimate the pose of the robot in environments with laser scan ambiguity.

The remainder of this paper is organized as follows. Section 2 describes the overall method for the indoor robot to minimize robot pose error using a graph structure-based SLAM from multi-sensors. To verify the superiority of the proposed method, we describe experimental environments and the overall system and present experimental results obtained with our approach in Section 3. In Section 4, conclusions and future work are discussed.

## 2. Graph Structure-Based SLAM Using Multi-Sensors

This section introduces our method in detail. First, GMapping [4], a grid-based SLAM with Rao–Blackwellized particle filters and the formulation of graph-based SLAM are described briefly. We then explain in detail how to fuse the feature data extracted from the monocular camera and depth data from the laser scanner for robot localization. Finally, we describe the method of pose graph generation and optimization.

### 2.1. Grid-Based SLAM with Rao–Blackwellized Particle Filters

GMapping is grid-based SLAM using Rao–Blackwellized particle filters. The particle depletion, a chronic problem of SLAM based on particle filters, was solved by using adaptive re-sampling in the GMapping. GMapping decreases the uncertainty of the robot pose by employing an approach called improved proposal distribution. This approach generates a proposal distribution based on the most recent sensor measurement assuming that the laser scans are more accurate than the odometry. This is a probabilistic method for estimating the robot pose, and it is possible to estimate the pose of the robot on a 2D plane by using depth data of a laser scanner. The GMapping algorithm can be utilized when the successive laser scans can be matched without ambiguity. However, robot pose error might occur if the GMapping algorithm is exploited in environments with laser scan ambiguity. It is therefore necessary to cope with this situation, and we describe graph-based SLAM for multi-sensor fusion in the next subsection.

### 2.2. Modeling of Graph-Based SLAM

In this subsection, we describe the general graph-based SLAM formulation and solution of graph-based SLAM [27]. A general graph-based SLAM can be written as follows using a conditional probability:

(1) $$p\left(x|z\right)\propto \prod_{i}p\left(z_{i}|x\right)$$

where *x* denotes the robot's state (or pose), *z* = {*z*_1_, ⋯ , *z_n_*}, *z_i_* denotes a measurement from a sensor at the *i*-th step and *p*(*z*_1_|*x*) is a potential function. The potential function for the measurement of each state is represented as follows:

(2) $$p\left(z_{i}|x\right)=\exp\left(-\frac{1}{2}r_{i}^{T}\Lambda_{i}r_{i}\right)$$

where Λ*_i_* denotes the covariance of the measurement *z_i_* and *r_i_* is the residual obtained by the difference between the prediction *f_i_* and the measurement *z_i_* as follows:

(3) $$r_{i}=f_{i}\left(x\right)=z_{i}$$

in general, the state prediction is nonlinear, and therefore, to simplify the problem, the prediction can be approximated as a first-order using Taylor series as follows:

(4) $$r_{i}\approx {\text{J}}_{i}\Delta x+f_{i}\left(x_{0}\right)-z_{i}$$

where J*_i_* is the Jacobian of *f_i_* with respect to *x* and *x*_0_ is the differentiating point. The solution to Equation (4) can be obtained by using the MLE (maximum likelihood estimation) method. After taking the logarithm of Equation (1), the solution is obtained by the following equation:

(5) $$x^{*}=\arg\underset{x}{\max}\left(-\frac{1}{2}\sum_{i}r_{i}^{T}\Lambda_{i}r_{i}\right)$$

the above equation is a least squares problem, and it is possible to estimate the value of x^*^ by using various optimization techniques.

### 2.3. Hybrid Method of a Monocular Camera and a Laser Scanner

**Figure 1:**
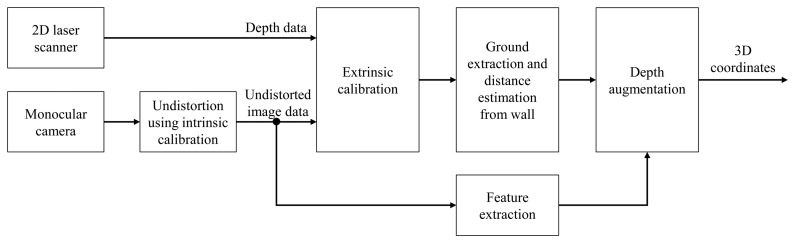
3D coordinate estimation of the camera feature by the hybrid method of a monocular camera and a laser scanner. Depth data and image feature points are extracted from the 2D laser scanner and the monocular camera. 3D coordinates of features are generated using the hybrid method by assuming that the wall is not inclined.

**Figure 2:**
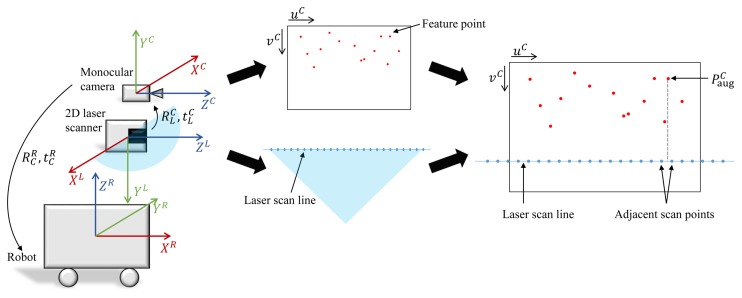
The concept of the hybrid method. Coordinate systems of the monocular camera, the 2D laser scanner and the robot are shown. The depth value of the feature point can be acquired from the depth value of the laser scan point on the same *u^C^*.

In this subsection, the method for fusing the feature data of the camera and the depth information of the laser scanner is described. The 3D robot pose is predicted using a hybrid method, and this information serves as the constraints of the graph-based SLAM. The overall algorithm and concept are shown in Figures 1 and 2, respectively. Before performing the hybrid method of the laser scanner and the monocular camera, intrinsic calibration is necessary in order to determine the intrinsic parameter of the camera. It is also necessary to know the relative pose between the camera and the laser scanner for fusing data from them. Therefore, the relative pose information between the two sensors is obtained through extrinsic calibration.

The general pinhole camera model is used in the subsequent mathematical derivation [28]. A 3D point *P^C^* = [*x^C^*,*y^C^*,*z^C^*]*^T^* in the camera coordinate system can be normalized to $P_{n}^{C}={\left[x_{n}^{C},y_{n}^{C}\right]}^{T}={\left[x^{C}/Z^{C},y^{C}/z^{C}\right]}^{T}$, and its corresponding 2D point *p^C^* = [*u^C^*, *v^C^*]*^T^* in the image plane is represented by the following equation:

(6) $$\left[\begin{array}{c} p^{C}\\ 1 \end{array}\right]=K^{C}\left[\begin{array}{c} p_{n}^{C}\\ 1 \end{array}\right]$$

where *K^C^* is the camera's intrinsic parameter, defined as follows:

(7) $$K^{C}=\left[\begin{array}{ccc} f_{x}^{C} & \alpha^{C}f_{x}^{C} & u_{0}^{C}\\ 0 & f_{y}^{C} & v_{0}^{C}\\ 0 & 0 & 1 \end{array}\right]$$

where $f^{C}={\left[f_{x}^{C},f_{y}^{C}\right]}^{T}$ is a focal length, ${\left[u_{0}^{C},v_{0}^{C}\right]}^{T}$ a principal point and *α^C^* a skewness of the camera. These values are acquired from intrinsic camera calibration. Since the skewness of most cameras is zero, the image coordinates can be written as follows:

(8) $$u^{C}=f_{x}^{C}\frac{x^{C}}{z^{C}}+u_{0}^{C}$$

(9) $$v^{C}=f_{x}^{C}\frac{y^{C}}{z^{C}}+v_{0}^{C}$$

Equations (8) and (9) can be reformulated as follows:

(10) $$x^{C}=\frac{\left(u^{C}-u_{0}^{C}\right)z^{C}}{f_{x}^{C}}$$

(11) $$y^{C}=\frac{\left(v^{C}-v_{0}^{C}\right)z^{C}}{f_{y}^{C}}$$

the laser scan data per scan contain the scan angle *θ* and depth information *d* at the angle *θ*. Referring to the coordinate system in Figure 2, a 3D point *P^L^* = [*x^L^*, *y^L^*, *z^L^*]*^T^* in the laser scanner coordinate system can be written as follows:

(12) $$P^{L}=\left[\begin{array}{c} d\cos\theta \\ 0\\ d\sin\theta \end{array}\right]$$

in Equation (12), the vertical component of the laser scan point is zero, since only the horizontal component can be measured from the 2D laser scanner. The 3D point *P^L^* can be transformed to a point in the camera coordinate system using coordinate transformation from the laser scanner to the camera as follows:

(13) $$\left[\begin{array}{c} P^{C}\\ 1 \end{array}\right]=\left[\begin{array}{cc} R_{L}^{C} & t_{L}^{C}\\ 0 & 1 \end{array}\right]\;\left[\begin{array}{c} P^{L}\\ 1 \end{array}\right]$$

where $R_{L}^{C}$ is the rotation matrix and $t_{L}^{C}$ is the translation vector from the laser scanner coordinate system to the camera coordinate system. This transformation can be acquired through extrinsic calibration from the laser to the camera. In Figure 2, if the laser scan line is overlaid onto the image, 3D points of the laser scan line in the camera coordinate system can be obtained from Equation (13).

Let us assume that the wall is exactly normal to the ground and vertically flat. Then, all points on the same *u^C^* on the image plane will have the same depth value. The depth value of the feature point on the image is determined using a point on the overlaid laser scan line having the same *u^C^*. Since the points on the overlaid laser scan line are discrete, the corresponding depth values can be acquired by linear interpolation of the depth values from adjacent overlaid scan points. Let us denote the linearly-interpolated depth value as *z̃*. By replacing *z^C^* in Equations (10) and (11) with *z̃*, the augmented 3D feature point in the camera coordinate system can be obtained as follows:

(14) $$P_{\text{aug}}^{C}=\left[\begin{array}{c} \frac{\left(u^{C}-u_{0}^{C}\right)\tilde{z}}{f_{x}^{C}}\\ \frac{\left(v^{C}-v_{0}^{C}\right)\tilde{z}}{f_{y}^{C}}\\ \tilde{z} \end{array}\right]$$

The 3D point *P^C^* in the camera coordinate system can be transformed to the robot coordinate point *P^R^* = [*x^R^*, *y^R^*, *z^R^*]*^T^* as follows:

(15) $$\left[\begin{array}{c} P^{R}\\ 1 \end{array}\right]=\left[\begin{array}{cc} R_{C}^{R} & t_{C}^{R}\\ 0 & 1 \end{array}\right]\;\left[\begin{array}{c} P^{C}\\ 1 \end{array}\right]$$

where $R_{C}^{R}$ is the rotation matrix and $t_{C}^{R}$ is the translation vector from the camera coordinate system to the robot coordinate system. This transformation also can be acquired using extrinsic calibration from the camera to the robot. Since it is possible to determine the height of the laser scan from the ground using these relative poses, the ground segment can be extracted from the image plane. The remaining portion can be regarded as the wall components, and it is assumed that the wall is vertical and not inclined. This assumption can be relieved later, because outliers can be removed through the feature matching process. The feature extraction algorithm is then run, and only feature points on the wall components in the 2D image are exploited in this algorithm.

**Figure 3:**
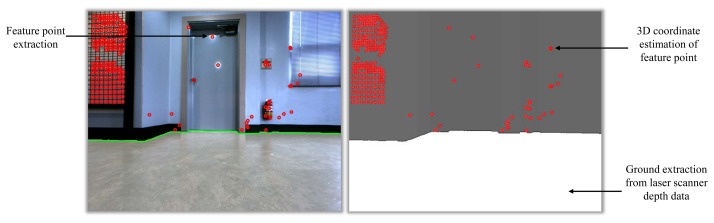
Extracted features on the wall in the image. Red circles indicate the extracted image feature points. The white region in the figure is the extracted ground. The gray region in the right side figure is the wall.

Figure 3 shows an example using the proposed algorithm. It is shown that the wall surface and the ground surface are separated accurately using the depth data of the laser scanner, and the distance to the wall surface is also estimated at the same time, which enables estimation of the 3D coordinates of feature points extracted from the image.

### 2.4. Pose Data Acquired from Multi-Sensors

**Figure 4:**
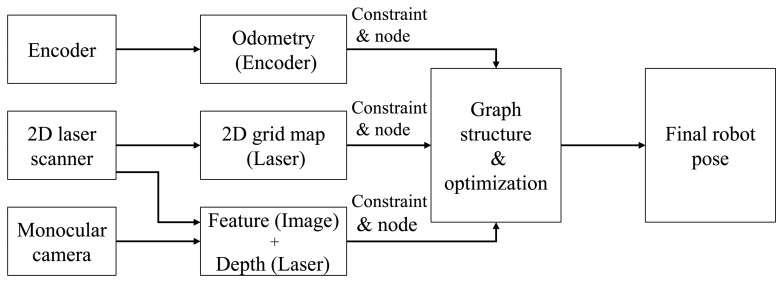
Graph structure generation from respective sensor data. The constraints and nodes are generated from the prediction from each sensor. The final pose of the robot is estimated using graph optimization.

After installing multi-sensors on the robot, the pose of the robot is estimated using the respective sensors, and then, a hybrid algorithm fuses the respective results, as shown in Figure 4. The covariance values and the measurement values from each sensor are used for generating the constraints of the graph structure. The final corrected robot pose is obtained by graph optimization by organizing the graph structure using the generated constraints information. Odometry information is generated by encoders, and this information is used for generating the pose constraint. A 2D grid map is made using depth data from the 2D laser scanner. The 2D robot pose constraint is then generated from the 2D grid map and ICP matching using GMapping [4]. After extracting the feature points by the SURF (speeded up robust features) [29] algorithm from a camera image, the 3D coordinate of each feature point is obtained by augmenting the depth information from the 2D laser scanner. The 3D robot pose constraint is then generated from the 3D coordinates of the feature points. The hybrid method of obtaining the robot pose using a monocular camera and a laser scanner was described in detail in the previous subsection.

**Figure 5:**
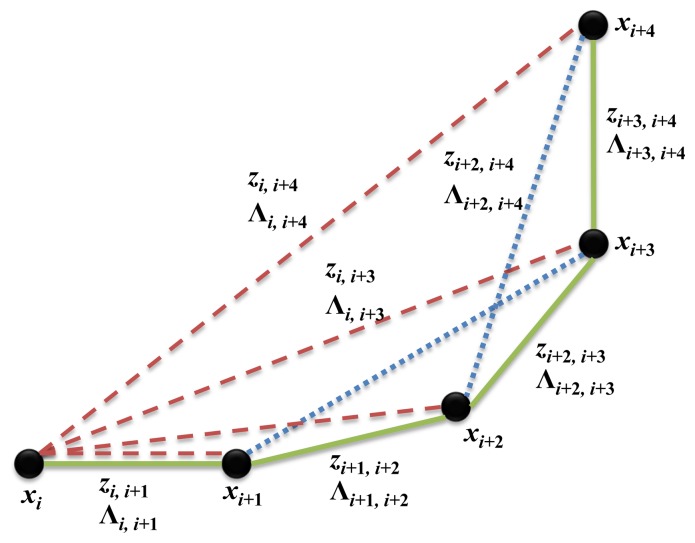
Example of a graph structure. The black node is the robot pose, and the edge of the graph structure represents the constraint between nodes. The green solid line is the constraint from the odometry; the red dashed line denotes the constraint from the 2D laser scanner data; and the blue dotted line represents the constraint from the hybrid method of the monocular camera and the laser scanner.

The example of generating the pose graph structure from multi-sensors is shown in Figure 5. Each node represents the pose of the robot (*x_i_*), and the edge that is connecting nodes is the constraint, which consists of a measurement (*z_i_*_,_*_j_*) and its covariance (Λ*_i_*_,_*_j_*). The green solid line denotes the constraint created using dead-reckoning from the odometry. The red dashed line represents the constraint resulting from the laser scanner using GMapping [4]. In using GMapping, a 2D grid map is obtained from the scan matching method using a 2D laser scanner. It is then possible to acquire the constraint using Rao–Blackwellized particle filters. Since GMapping is the particle filter-based SLAM algorithm, it only provides the current pose estimate relative to the origin. Therefore, the constraints between successive nodes in the graph structure cannot be generated using GMapping, and only the constraints between the origin and the robot can be generated. The blue dotted line represents the constraint generated using the hybrid method from the feature points of the camera and the depth data of the laser scanner. Only if the loop closure is detected, the hybrid method generates constraints between the corresponding nodes. Therefore, the constraint may not be generated for successive nodes by the hybrid method. The pose prediction from each sensor and the covariance values are used as constraints in the graph to correct the final robot pose estimation using a graph optimization technique.

### 2.5. Pose Graph and Optimization

**Figure 6:**
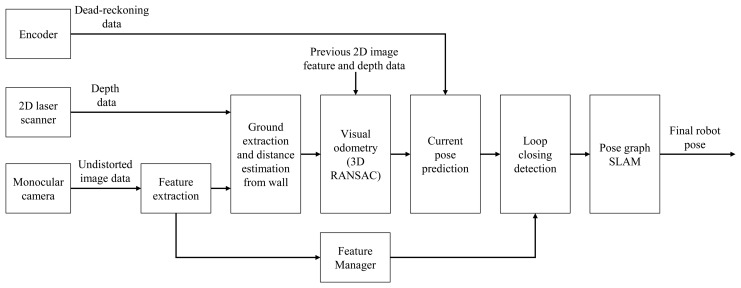
Flow diagram of the overall algorithm. For the final robot pose, predictions using the sensor fusion method are exploited. The robot pose is predicted using dead-reckoning of the encoder and is updated using feature matching through loop closure detection.

This subsection explains the method of graph structure construction by generating nodes and constraints from the depth data of the laser scanner and the feature points from the camera. The basic SLAM algorithm used in this method is based on the 3D SLAM algorithm [13], and only a monocular camera and a laser scanner are used to estimate the distance information of the feature points. A flow diagram of the overall algorithm is shown in Figure 6. When data from the monocular camera are inputted, the feature points are extracted from the 2D image. The extracted feature points on the wall components are used for visual odometry and in the loop closure detection. 3D coordinates of the feature points on the image are obtained using the hybrid method described in the previous subsection. The 3D pose (six DoF (degrees of freedom)) between matched images is determined with 3D-RANSAC [26] using 3D coordinates of the matched feature points on the paired image. The initial connection between the nodes uses the odometry information. The 2D image feature point obtained at each node is stored in the feature manager and used for constructing the constraint between nodes. The feature manager finds the previous node that has a common image feature, using the feature data of the newly-generated node. This process is called loop closure detection [13]. The constraint between nodes imposed by using the feature points is used as information for forming a graph structure representing the entire trajectory of the robot. The pose graph SLAM is then applied to the graph structure formed via the above-described method to obtain the final corrected pose. The graph-based SLAM problem is solved by optimizing the graph model with sparse linear algebra techniques in real time [2].

## 3. Experimental Results

In this section, we present the experimental setup and results for the graph structure-based SLAM method using multi-sensors. To validate the proposed method, the overall system, the experimental environment, the method for implementation and the calibration results are described below. The experimental results obtained in feature-poor laser observation environments are then illustrated. In order to demonstrate the performance of our method, numerical results are presented through a comparison with GMapping in terms of the pose error in the whole robot trajectory.

### 3.1. Experimental Setup

**Figure 7:**
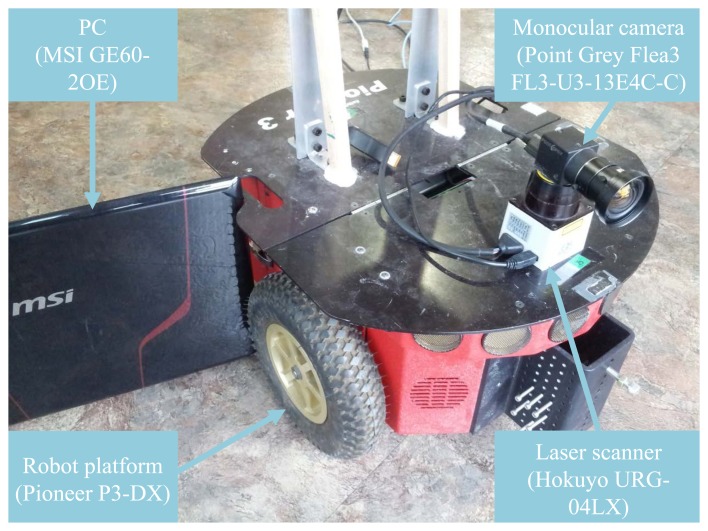
Experimental system. The system consists of Pioneer P3-DX, Hokuyo URG-04LX, Point Grey Flea3 FL3-U3-13E4C-C and MSI GE60-2OE.

The overall system for the experiment was configured as follows. Pioneer P3-DX [30] was used as the robot platform, and Hokuyo URG-04LX [31], a laser scanner, Point Grey Flea3 FL3-U3-13E4C-C [32], a monocular camera, and MSI GE60-2OE [33], a laptop PC, were installed on the robot platform. The whole system is illustrated in Figure 7. The specifications of each device are described in Table 1. The Pioneer P3-DX provides wheel odometry information through 100 tick encoders. The Hokuyo URG-04LX has a maximum range of about 4 m. Although the update rate of the Flea3 camera is 60 Hz, the image processing procedure is carried out only when a node of the graph structure is added for real-time computation. The MSI GE60-20E PC is capable of GPU (graphics processing unit) computing for fast image processing. The overall framework was implemented on a Linux platform, Ubuntu 12.04, based on an open source and open library, including OpenCV (Open Computer Vision) 2.4 [34], ROS (Robot Operating System) hydro [35] and iSAM (incremental Smoothing And Mapping) [2].

**Figure 8:**
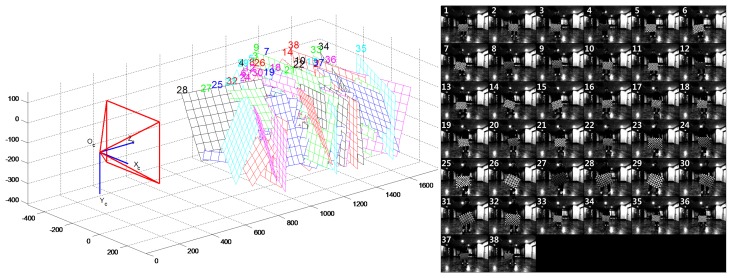
Camera's intrinsic calibration results. The left side of the figure is the 3D geometric results of the checker board from the camera calibration, and the right side of the figure is the checker board images used for calibration.

Calibration was performed for the hybrid camera and laser scanner approach. In order to obtain the camera's intrinsic parameters, intrinsic calibration was conducted by using Zhang's method [36,37]. This calibration method was implemented with an open source technique called the Camera Calibration Toolbox [38]. A total of 38 checker board images were used to perform the camera's intrinsic parameter calibration. The pattern of the checker board consists of 13 vertical by 10 horizontal lines, and the grid size is 41.3 mm × 41.3 mm. Figure 8 shows the intrinsic parameter calibration results. The right side of Figure 8 is the image dataset for camera calibration, and the left side shows the 3D geometric results of the checker board obtained using the intrinsic parameters from the camera calibration. The camera's intrinsic parameters are listed in Table 2, and are used for constructing *K^C^* in Equation (7).

In order to obtain the 3D coordinates of the feature points from the hybrid laser scanner and camera approach, it is necessary to know the relative pose between these sensors. This entails extrinsic parameter calibration, and the relative pose is determined using Zhang and Pless's method [39]. A total of 38 datasets were exploited for the extrinsic parameter calibration, and the results are as follows:

(16) $$R_{L}^{C}=\left[\begin{array}{ccc} -0.9997 & -0.0245 & -0.0052\\ 0.0247 & -0.9985 & -0.0483\\ -0.0041 & -0.0484 & 0.9988 \end{array}\right]$$

(17) $$t_{L}^{C}=\left[\begin{array}{c} -0.0210\\ -0.0108\\ -0.0189 \end{array}\right]$$

**Figure 9:**
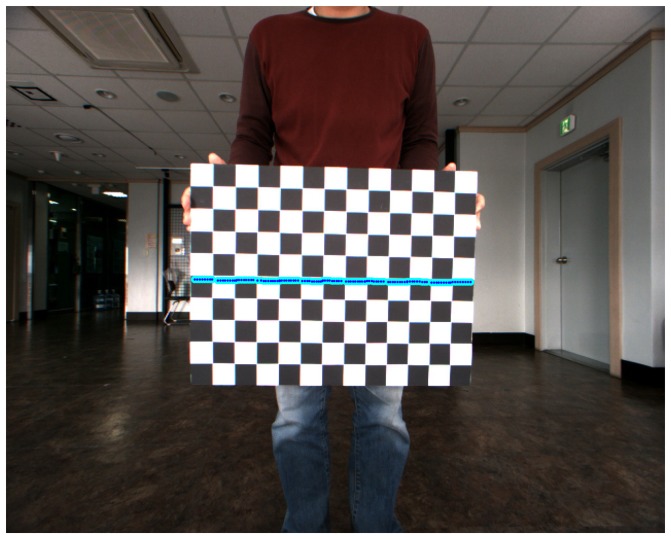
Projection of the laser scanner's depth data to the checker board image.

The projected depth data from the laser scanner on the image using the parameters are shown in Figure 9. The laser scanner's depth data for the checker board are overlaid on the image plane. Figure 9 shows that the calibration is correct, as the checker board image and the projected depth data from the laser scanner match well.

In addition, to acquire the robot pose from the results of the hybrid method, the relative transformation between the camera and the robot should be determined. A similar method to that presented in our previous study [40] is used to obtain extrinsic parameter calibration results. A marker whose size is known in advance is measured through the camera installed on the robot by our previous research [40] to get six DoF displacement from the camera to the marker. Additionally, the distance from the robot to the marker is measured assuming that the robot is placed directly in front of the marker to acquire six DoF displacement from the robot to the marker. Then, using a homogenous transformation, it is possible to calculate the extrinsic parameter calibration results. The numerical results of the extrinsic calibration between the camera and the robot are as follows:

(18) $$R_{C}^{R}=\left[\begin{array}{ccc} 0.0675 & -0.0695 & 0.9953\\ -0.9976 & 0.0077 & 0.0682\\ -0.0124 & -0.9976 & -0.0688 \end{array}\right]$$

(19) $$t_{C}^{R}=\left[\begin{array}{c} 0.0040\\ -0.4029\\ -0.1553 \end{array}\right]$$

**Figure 10:**
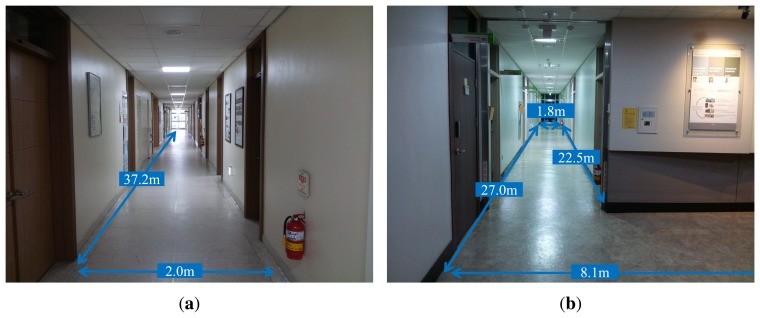

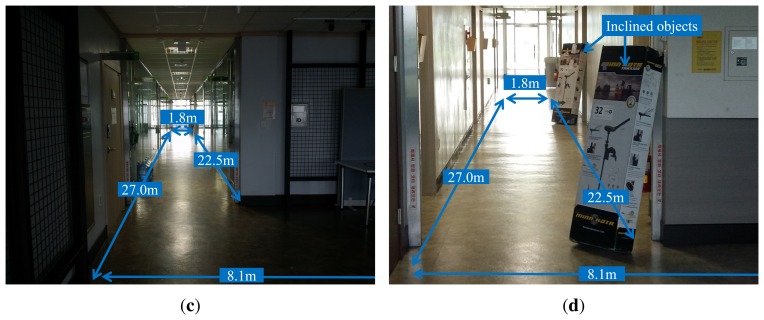
Experimental environment. Dataset 1 is a long corridor. Datasets 2, 3 and 4 are L-shaped, and some parts of the wall in Dataset 3 are glasses. Datasets 2 and 4 are the same place. However, two inclined objects are placed in Dataset 4. (**a**) Dataset 1; (**b**) Dataset 2; (**c**) Dataset 3; (**d**) Dataset 4.

**Figure 11:**
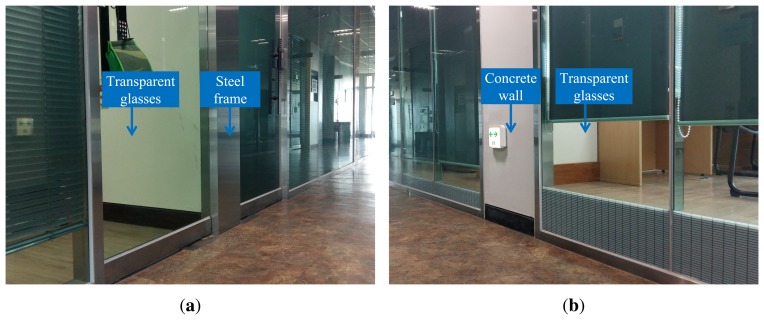
Snapshots of Dataset 3. Some parts of the wall are made of transparent glass. However, parts of the wall seen within the field of view consist of a steel frame or a concrete wall. (**a**) Steel frame with transparent glasses; (**b**) concrete wall with transparent glasses.

**Figure 12:**
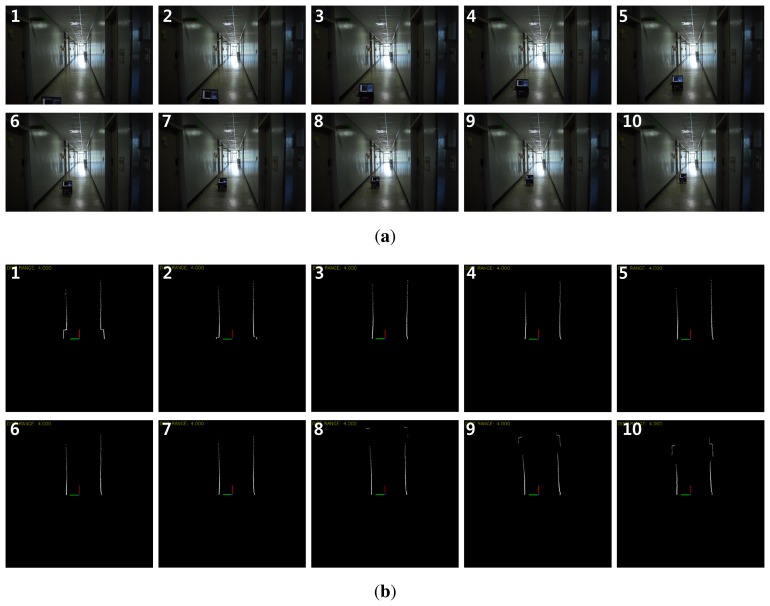
Scene examples of the laser scan ambiguity. From the third to seventh scenes, the ambiguity occurs because the laser scanner measures only straight wall components. (**a**) Snapshots of robot for every 0.45 m of movement; (**b**) Laser scan data corresponding to each robot position.

Figure 10 shows the experimental environment to verify the proposed method. The experimental environments for Datasets 1, 2, 3 and 4 are shown in Figure 10. The size of a long corridor in Dataset 1 is 36.4 m × 2.0 m. The laser scanner's depth data of this space have almost the same values because of the shape of this environment. Therefore, in this space, localization using the laser scanner provides good performance for the lateral direction. However, the localization results along the moving direction are not reliable. The number of ground truth positions in Dataset 1 is 85. The size of the L-shaped space in Dataset 2 is 16.0 m × 0.9 m, and the detailed dimensions are shown in Figure 10. The experimental environment of Dataset 2 is a long corridor similar to that of Dataset 1, except there is a corner. The number of ground truth positions in Dataset 2 is 43. The size of the L-shaped space in Dataset 3 is 18.0 m × 0.9 m, and some parts of the wall are made of transparent glass. In experimental environments where the wall contains the transparent glasses, there is a high probability that a laser light passes through the glass or is diffused by the glass. However in Dataset 3, parts of the wall seen within the field of view consist of a steel frame or a concrete wall. The snapshots of Dataset 3 are shown in Figure 11. Although laser scan ambiguity still occurs partially, it is possible to estimate the robot pose information by measuring the laser depth data from these components. The number of ground truth positions in Dataset 3 is 43. The experimental conditions and the location of Dataset 4 is the same as Dataset 2. However, obstacles, which are not normal to the ground, but inclined, are placed in Dataset 4 to verify whether the proposed method robustly estimates the robot pose or not. Ground truth positions are acquired by a manual measurement thanks to the regular grids on the floor. In order to derive experimental results, the robot passed through the ground truth positions manually measured in advance. Despite the structure of the doors and the edges, the laser scan ambiguity occurs in the experimental environment; since the maximum detectable range of the laser scanner is limited (about 4 m in our experiments), these components cannot be measured, and the laser scanner acquires uniform data from the wall. The scene examples of the laser scan ambiguity are shown in Figure 12, where the successive number indicates a snapshot of robot and laser scan data corresponding to each robot position for every 0.45 m of travel of the robot. The third to seventh scenes in Figure 12 exhibit the laser scan ambiguity.

### 3.2. Results

**Figure 13:**
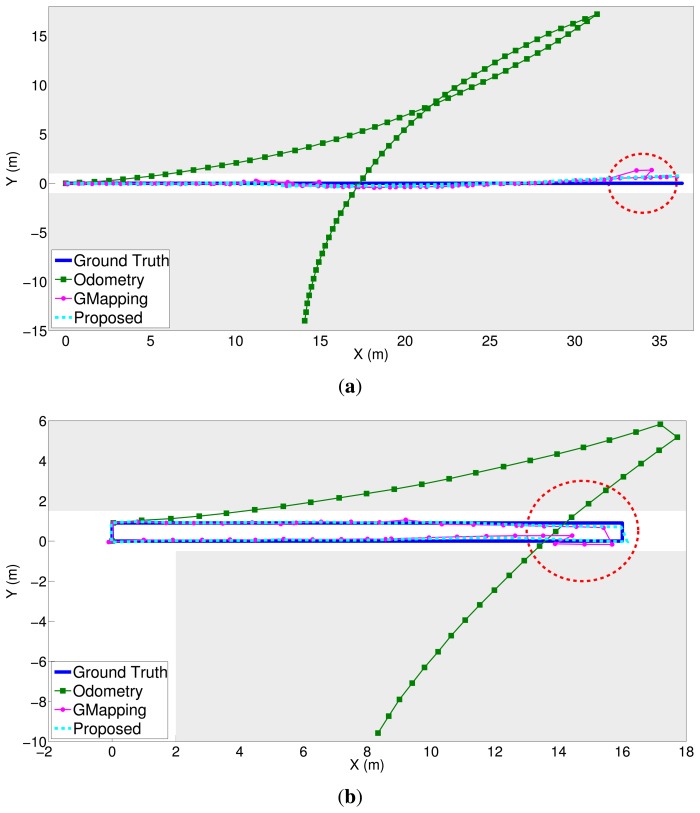

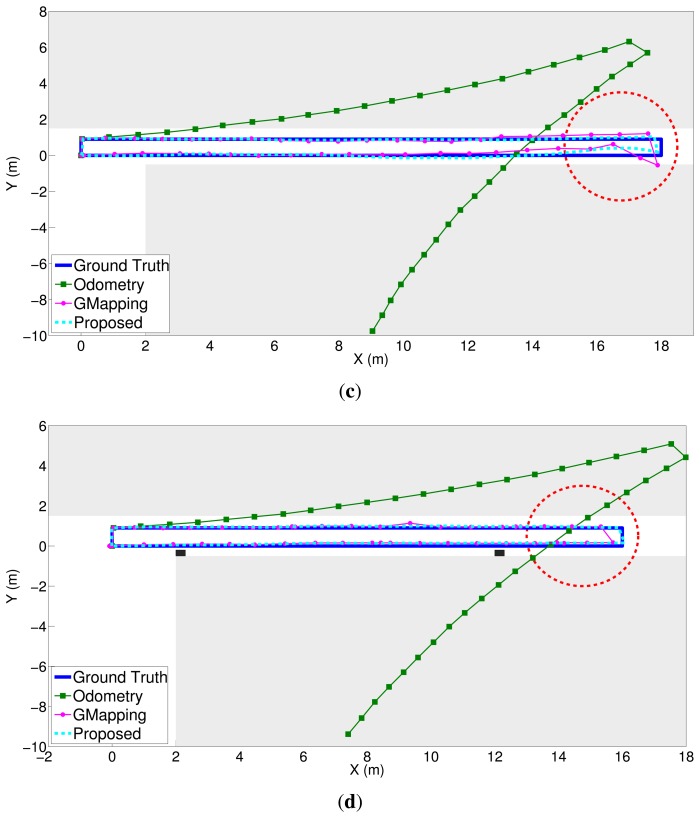
Experimental results comparing the odometry, GMapping and the proposed method. The trajectory of the proposed method is almost the same as the ground truth. Gray regions depict the walls. (**a**) Dataset 1; (**b**) Dataset 2; (**c**) Dataset 3; (**d**) Dataset 4.

**Figure 14:**
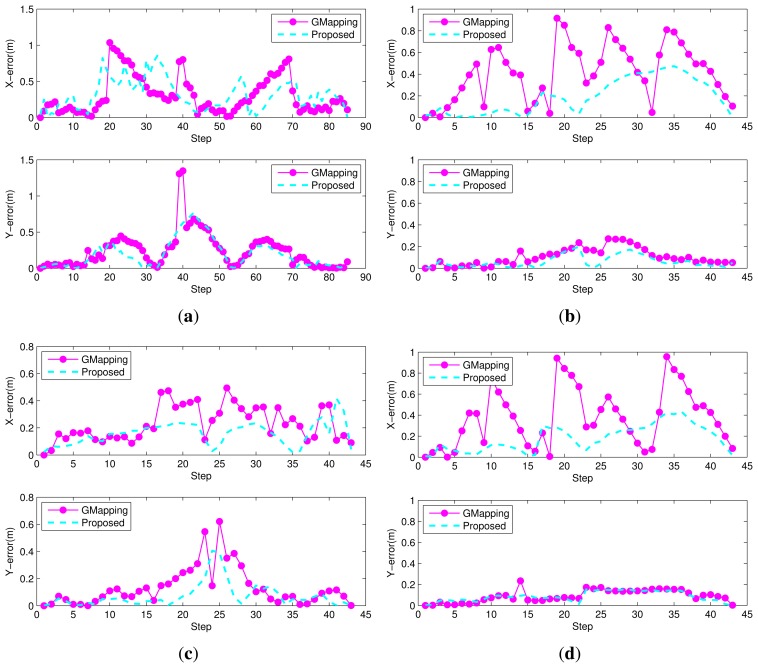
Position error at each time step for GMapping and the proposed method. (**a**) Dataset 1; (**b**) Dataset 2; (**c**) Dataset 3; (**d**) Dataset 4.

Figures 13 and 14 show the comparison results. In Figure 13, the blue solid line indicates the ground truth value, the green rectangle solid line the trajectory from the robot's odometry, the magenta circle solid line the results obtained from GMapping [4] and the cyan dotted line the results obtained by the proposed algorithm. In Figure 14, the magenta circle solid line means the error from GMapping [4] and the cyan dashed line the error by the proposed algorithm. The largest position error is found in the robot odometry, where only the information obtained from the encoders of the robot is used. The odometry error caused by the slip effect of the robot wheel gradually accumulates. The results obtained by GMapping are similar to the ground truth, but there is some error. Because the depth values scanned from the laser scanner are uniform in a corridor, it is not easy to compensate for the errors from odometry through GMapping. In particular, in environments such as Dataset 1, it is confirmed that the error is large.

GMapping shows significant localization errors, as indicated by the circled regions in Figure 13. This can be explained as follows. In environments with laser scan ambiguity, such as a long corridor, the output of the laser scanner's depth data is uniform. The lack of features from laser scan depth data leads to wrong scan matching results. However, accurate pose estimation is possible through the proposed method, because the 3D coordinates of feature points by the hybrid method remove feature ambiguity. Even if loop closure occurs, it is difficult to estimate the whole robot trajectory with GMapping, because this particle-based SLAM approach cannot retain the past entire robot pose information. If GMapping detects loop closure correctly by measuring the same features previously scanned, then the correction of the robot pose can occur in a different direction to the odometry, for example around the region X = 14 m, in Figure 13b. Through loop closure and graph optimization, the proposed method enables the whole robot trajectory to be estimated correctly, and the localization error is significantly reduced.

To confirm the localization performance of the proposed algorithm numerically, the RMSE (root mean square error) is presented in Table 3. The position error was determined using the measured ground truth values along the path. The error from odometry, as shown in Figure 13, is the largest in all datasets with respect to the x-axis and y-axis directions. The error from GMapping is smaller than that of odometry. The error of the proposed method, meanwhile, is the smallest along the x-axis and y-axis. It can therefore be confirmed that the position estimation using the proposed method is the closest to the ground truth. As mentioned before, it is found from the numerical results of Table 3 and Figure 14 that the lateral direction (Y direction) performance from GMapping is generally better than the longitudinal direction in environments such as the long corridor. Despite obstacles that do not fit the assumptions made, the results of the proposed method in Dataset 4 are numerically similar to those of Dataset 2. There is less laser scan ambiguity due to the obstacles; however, laser scan ambiguity still occurs due to the wall components of the long corridor. The error of GMapping in Dataset 4 is smaller than in Dataset 2. However, the error of GMapping is larger than the proposed method both in Datasets 2 and 4. Therefore, the proposed method robustly estimates the robot pose, because the features on the inclined obstacles are rejected in the process of feature matching. The proposed algorithm has a small computational burden, because image processing is carried out only when the node is added. The node is generated for every 0.5 m of travel or for every 20 degrees of rotation. In addition, the SURF feature extraction algorithm is accelerated by the GPU for real-time operation. The average update rate of the overall algorithm is 20 Hz, which is sufficient for real-time operation.

## 4. Conclusions

In this paper, we proposed a novel localization algorithm using graph-based SLAM for environments with laser scan ambiguity where the depth information of the laser scanner is constant, such as a long corridor. For the proposed method, we fused a monocular camera and a laser scanner using a graph structure. The proposed algorithm was verified by conducting experiments and comparing the results with those from GMapping. The error of the proposed method was smaller than that of GMapping. The inclination of the wall does not affect the results of the proposed method, because feature outliers on the inclined wall are rejected via the feature matching algorithm. The proposed method is thus useful for localization in indoor environments with laser scan ambiguity, such as a long corridor. For future work, the localization method will be extended by applying a probability model for determining the 3D coordinates of the feature points in an outdoor environment.

## Figures and Tables

**Table 1:** System specifications.

**Equipment**	**Specifications**	
Robot platform (Pioneer P3-DX)	Maximum forward speed	1.2 m/s
	Maximum angular speed	300 deg/s
	Operating payload	17 kg

Laser scanner (Hokuyo URG-04LX)	Range	60 mm–4095 mm
	Range resolution	1 mm
	Field of view	240 deg
	Angular resolution	0.36 deg
	Update rate	10 Hz

Monocular camera (Point Grey Flea3 FL3-U3-13E4C-C)	Resolution	1280 × 1024 pixels
	Update rate	60 fps
	Megapixels	1.3 MP

PC (MSI GE60-2OE)	CPU	Intel Core i7-4700MQ (2.4 GHz)
	GPU	Nvidia GeForce GTX 765 M (2 GB GDDR5)

**Table 2:** Intrinsic parameter calibration results. The camera lens distortion *k^C^* is a vector containing tangential and radial distortion coefficients [36], and the camera pixel error *e^C^* denotes a vector of the standard deviation of the reprojection error along the *u^C^* and *v^C^* axes.

	**Value**
Focal Length	$\left[f_{x}^{C},f_{y}^{C}\right]=\left[693.8864,696.4908\right]$
Principal point	$\left[u_{0}^{C},v_{0}^{C}\right]=\left[656.9713,513.0494\right]$
Skewness	*α^C^* = [0.0000]
Distortion	*k^C^* = [−0.0465, 0.1098, −0.0034, 0.0034, 0.0000]
Pixel error	*e^C^* = [0.26082, 0.27740]

**Table 3:** RMSE results. The error of the odometry is the largest. The error of GMappingis smaller than that of the odometry, and the error of the proposed method is the smallest with respect to Datasets 1, 2, 3 and 4.

**RMSE** (Unit: m)	**Odometry**		**GMapping**		**Proposed Method**	
		
	X	Y	X	Y	X	Y
Dataset 1	4.5610	9.0247	0.4011	0.3392	0.3807	0.2749
Dataset 2	3.5888	3.8041	0.4885	0.1295	0.2499	0.0807
Dataset 3	3.3805	3.9649	0.2640	0.1905	0.1791	0.1140
Dataset 4	3.3052	3.6068	0.4648	0.1064	0.2190	0.0978
